# Analysis of magnetic resonance imaging features of ovarian thecoma

**DOI:** 10.1097/MD.0000000000020358

**Published:** 2020-05-22

**Authors:** Zhi Li, Qingwei Hu, Zhiqin Luo, Zaixing Deng, Wei Zhou, Linghong Xie

**Affiliations:** a Department of Radiology; b Department of Obstetrics and Gynecology; c Department of Pathology, Huzhou Maternity & Child Health Care Hospital; d Department of Radiology, Huzhou Central Hospital, Huzhou, Zhejiang Province, China.

**Keywords:** diagnosis, magnetic resonance imaging, ovarian neoplasms, ovarian thecoma

## Abstract

To investigate the magnetic resonance imaging (MRI) findings in ovarian thecoma and improve preoperative diagnostic accuracy.

Retrospective analysis was performed on 45 patients with surgically and pathologically confirmed ovarian thecoma. Patients were grouped into those with maximum lesion diameter ≥5 cm and <5 cm. Diagnostic scores (up to 6 points) were evaluated on the basis of MRI performance.

The ≥5 cm group contained 36 cases (cystic necrosis, 32 cases) with the following findings: T_1_WI: isointense signal, 22 cases; slightly hypointense signal, 14 cases; T_2_WI: isointense signal, 6 cases; slightly hypointense signal, 21 cases; slightly hyperintense signal, 9 cases; Diffusion-weighted imaging (DWI): hyperintense signal, 23 cases; mixed hyperintense signal, 13 cases; slight enhancement on dynamic enhanced scans; pelvic fluid accumulation, 31 cases. The diagnostic score evaluations yielded 6 points in 31 cases, 5 points in 1 case, 4 points in 2 cases, and 3 points in 2 cases. The <5 cm group contained 9 cases (cystic necrosis, 3 cases) with the following findings: T_1_WI: isointense signal, 3 cases; slightly hypointense signal, 6 cases; T_2_WI: isointense signal, 2 cases; slightly hypointense signal, 4 cases; slightly hyperintense signal, 3 cases; DWI, hyperintense signal; slight enhancement in 8 cases and significant enhancement in 1 case; pelvic fluid accumulation, 4 cases. The diagnostic score evaluations yielded 6 points in 3 cases, 5 points in 1 case, 4 points in 4 cases, and 3 points in 1 case. (iii) Incidence of pelvic fluid accumulation and cystic necrosis differed depending on the size of the lesion (*P* = .007, .000).

Larger lesions show hyperintense or mixed hyperintense signals on DWI along with pelvic fluid and cystic necrosis; whereas, smaller lesions show a hyperintense signal on DWI, cystic necrosis is rare. MRI characteristics along with the patient age and laboratory findings can improve the accuracy of preoperative diagnosis of these lesions.

## 1 Introduction

Ovarian thecoma is a tumor originating from the ovarian stroma, with a low incidence rate of about 0.15% to 1% of all ovarian tumors.^[1]^ Most of them are benign and malignancy is rare.^[2]^ The clinical manifestations of this tumor lack specificity, and preoperative diagnosis is difficult.^[3]^ MRI has better soft tissue resolution and can accordingly reflect the histological characteristics of ovarian thecoma and clearly show the size and shape of the lesion and its relationship with surrounding tissues; moreover, the accuracy of preoperative lesion localization diagnosis is high. However, the accuracy of qualitative diagnosis is low, and unfortunate misdiagnosis with other solid or cystic masses of the ovary may result in limited clinical support. The MRI images of 45 cases of ovarian thecoma were retrospectively analyzed. MRI image features and diagnostic points were designed to improve the understanding of the disease and accuracy of preoperative diagnosis.

## 2 Materials and methods

A retrospective analysis of 45 patients with complete data from January 2010 to May 2019 and confirmed by surgery and pathology as ovarian thecoma MRI images was conducted. The patients were from Huzhou Maternity & Child Health Care Hospital and Huzhou Central Hospital. All patients provided written informed consent to undergo MRI. Patient age ranged from 31 to 75 years, with a median age of 55 years. All were married and had been educated. Thirty six patients who had menopaused, ranging from 2 days to 1 year, were also included. The main clinical manifestations were as follows: 14 cases of postmenopausal vaginal bleeding, 12 cases of lower abdominal pain and discomfort, 19 cases of physical examination resulting in pelvic mass; 10 cases with elevated CA125, and 20 cases with elevated estrogen levels. Data cannot be shared publicly, because of the authors do not have written informed consent from the participants to share raw data. Thus, sharing the data would violate participant confidentiality.

### 2.1 MRI examination

All patients underwent pelvic plain and enhanced scans before surgery. MRI scans were performed using Siemens Avanto 1.5T MR and GE Signa 1.5T MR imagers, all using pelvic coils. The Siemens MR has a regular layer thickness of 5 mm; layer spacing, 1.5 mm; field of view (FOV), 380 mm × 380 mm; and number of excitations, 2. Horizontal axis T_1_WI: Repetition time (TR)/echo time (TE) 500 ms/12 ms; horizontal axis T_2_WI: TR/TE 4000 ms/85 ms; sagittal T_1_WI: TR/TE 550 ms/12 ms; sagittal Bit T_2_WI: TR/TE 4500 ms/85 ms; Coronal T_2_WI: TR/TE 4000 ms/100 ms; and Horizontal axis DWI: TR/TE 4000 ms/85 ms. The GE MR conventional layer thickness was 6 mm and layer spacing, 1 mm; FOV was 320 mm × 256 mm, and the number of excitations was 2. Horizontal axis T_1_WI:TR/TE 780 ms/11 ms; horizontal axis T_2_WI:TR/TE 4000 ms/85 ms; sagittal T_1_WI:TR/TE 780 ms/11 ms; sagittal position T_2_WI:TR/TE 4000 ms/85 ms; Coronal T_2_WI:TR/TE 4000 ms/85 ms; and Horizontal axis DWI:TR/TE 4000 ms/85 ms. The contrasting agent was docetaxel or omepramine administered as an IV bolus at the rate of 0.1 mmol/kg body weight.

### 2.2 MRI image analysis

All cases were imaged by 2 experienced deputy chief physicians in a double-blind manner. All disagreements were resolved by discussion. On the basis of the maximum diameter of the lesion, patients were divided into ≥5 cm and <5 cm groups.^[4]^ The following lesion characteristics were assessed:

1. location (left or right), size (maximum diameter), morphology (round, oval, or irregular shape), and boundary (clear or not);
2. cystic necrosis (range, morphology);
3. T_1_ WI, T_2_ WI plain scan findings (with reference to normal myometrial signals);
4. DWI features;
5. enhancement features (with reference to the division of the myometrium showing mild, moderate, and obvious enhancement); and
6. pelvic fluid.

### 2.3 Evaluation of MRI diagnostic score

Scores from 1 to 6 were assigned on the basis of the imaging findings of ovarian thecoma, with 1 point assigned for each of the following findings: single lesion, clear margin, combined cystic necrosis, hyperintense or mixed hyperintense signal on DWI, mild enhancement, and presence of pelvic fluid.

### 2.4 Pathological examination

Pathological analysis of all tissue specimens of this group of patients were carried out by a senior pathologist in our hospital.

### 2.5 Statistical methods

Statistical analysis was performed using SPSS 20.0 software. Data were expressed as mean ± standard deviation, analysis

1. There was with or without difference between the 2 groups of lesions and parts, pelvic fluid, menopause, and cystic necrosis. The χ^2^ test was used for comparisons between the groups, and statistical significance was set at *P* < .05.
2. In correlation analysis between elevated estrogen levels and cystic necrosis, which yielded r values, *P* < .05 indicated correlation between the 2 parameters.

## 3 Results

### 3.1 Location, size, shape, and boundary of the lesion

Forty five cases showed unilateral attachment lesions, with maximum lesion diameters of 3.0–18.0 cm and an average diameter of 9.7 ± 4.8 cm.

The ≥5 cm group included 36 cases (19 on the left side and 17 on the right side), with 22 cases showing a round shape, 9 showing an oval shape, and 5 showing an irregular shape; 34 cases showed a clear boundary while 2 cases showed an unclear boundary.

The <5 cm group included 9 cases (5 on the left side and 4 on the right side), with 5 cases showing a round shape, 3 showing an oval shape, and 1 showing an irregular shape (Table 1).

**Table 1:**
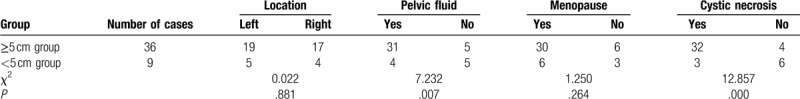
Lesion size and location, pelvic fluid, menopause, and cystic necrosis.

#### 3.1.1 Cystic necrosis

Thirty five cases showed cystic necrosis.

**Figure 1:**
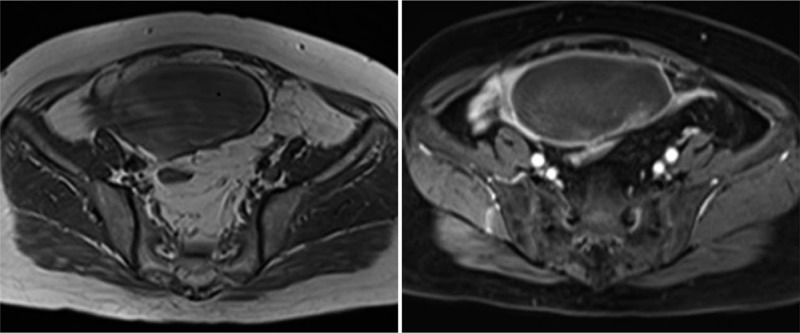
Left ovarian thecoma with massive hemorrhagic necrosis in a 69-year-old woman. (1-1) Cross-sectional T_1_WI image, with the scattered patchy slightly hyperintense signal shadow in the lesion indicating bleeding. (1-2) Slight enhancement of solid components after enhancement.

**Figure 2:**
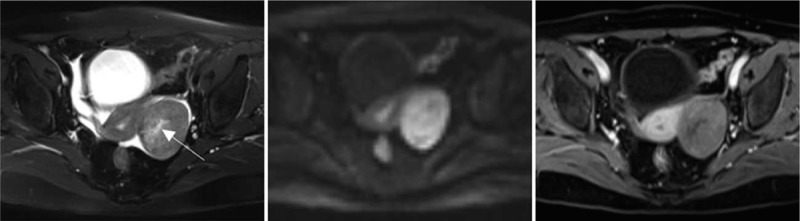
Left ovarian thecoma in a 68-year-old woman. (2-1) Cross-sectional T_2_WI showed that the lesion is located in the left posterior uterus. The edge is clear, elliptical, and a likely flocculent cyst change (arrowhead). A small amount of free effusion can be seen in the pelvic cavity. (2-2) B value of the DWI sequence of 1000 s/mm ^2^ showed a hyperintense signal with diffuse restriction and clear edges. (2-3) Enhanced scanning, with lesions showing uneven and mild enhancement; no enhancement was seen in the cystic zone.

1. In the ≥5 cm group, 32 cases showed large cystic necrosis with the following morphological distribution: hemorrhage, 1 case (Fig. 1); speckled, 2 cases; fissure, 2 cases; flocculent, 16 cases (Fig. 2); speckled merged fissure, 2 cases; speckled merged fissure, 6 cases; fissure-like flocculent, 2 cases; and round, 1 case.
2. In the <5 cm group, all 3 cases with cystic necrosis showed speckled morphology.
3. The relationship between elevated estrogen levels and lesion cystic necrosis is shown in Table 2.

**Table 2:**
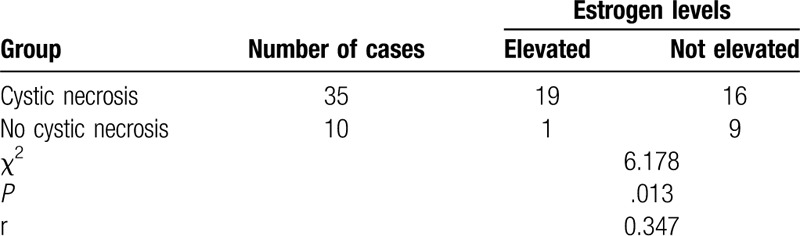
Analysis of the relationship between elevated estrogen levels and lesion cystic necrosis.

### 3.2 Characteristics of the solid component flat scan signal

(i) In the ≥5 cm group, T_1_WI showed an isointense signal in 22 cases and a slightly hypointense signal in 14 cases, while T_2_WI showed an isointense signal in 6 cases, a slightly hypointense signal in 21 cases, and a slightly hyperintense signal in 9 cases. (ii) In the <5 cm group, T_1_WI showed an isointense signal in 3 cases and a slightly hypointense signal in 6 cases, while T_2_WI showed an isointense signal in 2 cases, a slightly hypointense signal in 4 cases, and a slightly hyperintense signal in 3 cases.

### 3.3 DWI features

1. In the ≥5 cm group, 23 cases showed a hyperintense signal and 13 cases showed a mixed hyperintense signal.
2. In the <5 cm group, 9 cases showed a hyperintense signal.

### 3.4 Enhancement features

**Figure 3:**
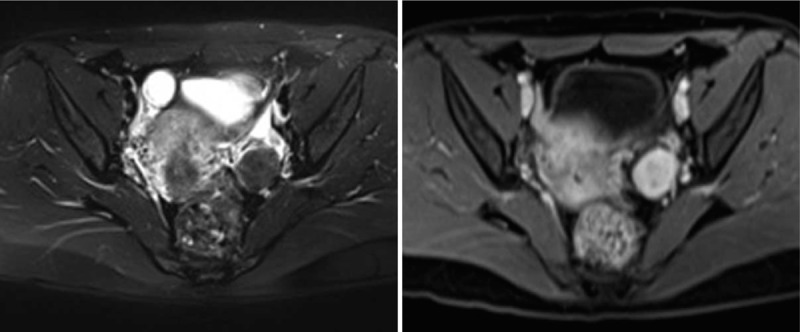
Left ovarian thecoma in a 32-year-old woman. (3-1) T2WI in cross-section. The lesion is located on the left side of the uterus with clear edges. (3-2) Obvious enhancement after injection of the contrast agent.

1. The ≥5 cm group included 36 cases showing dynamic enhancement of the lesions, with mild enhancement in the arterial phase, venous phase, and delayed phase.
2. The <5 cm group included 8 cases of dynamic enhancement of the lesions, with mild enhancement in the arterial phase, venous phase and delayed phase; 1 case showed dynamic enhancement in the arterial phase, venous phase, and delayed phase (Fig. 3).

### 3.5 Pelvic effusion

Pelvic effusion was noted in a total of 35 cases.

The ≥5 cm group included 31 cases showing different degrees of pelvic fluid, with 1 case showing a small amount of pleural effusion.

The <5 cm group included 4 cases with a small amount of pelvic fluid.

### 3.6 Evaluation of MRI diagnostic score

1. The ≥5 cm group showed 6 points in 31 cases, 5 points in 1 case, 4 points in 2 cases, and 3 points in 2 cases.
2. The <5 cm group showed 6 points in 3 cases, 5 points in 1 case, 4 points in 4 cases, and 3 points in 1 case.

### 3.7 Other findings

MRI examination also showed 9 cases of endometrial thickening, 6 cases of uterine fibroids, 5 cases of adenomyosis, 5 cases of ovarian cysts, and 1 case of endometrial cancer.

### 3.8 Pathological results

All patients underwent surgical resection, and multiple lesions were further processed for pathological examination. Thirty eight cases were pathologically diagnosed as being benign ovarian thecomas.

## 4 Discussion

Ovarian thecomas occur frequently in postmenopausal women^[3,5–7]^ but are rare before the age of 40 years.^[8]^ The average age of onset is 59 years,^[6]^ and only few are cancerous,^[9]^ as the reported malignancy rate is 1% to 5%.^[1,10]^ Most of the lesions are unilateral, with bilateral lesions being rather rare, and the lesions typically vary in size. When the tumor size is large, it can cause abdominal pain, abdominal distension, and pelvic mass. Some estrogens lead to complications such as vaginal bleeding, endometrial hyperplasia and breast pain. The incidence of postmenopausal patients in this group was 80.0% (36/45), and the median age was 55 years. All patients had unilateral masses and were pathologically confirmed to be benign. Fourteen patients had postmenopausal vaginal bleeding, 12 had lower abdominal pain and discomfort, 19 had a pelvic mass on physical examination. The CA125 antigen is a glycoprotein with a high molecular weight and can be recognized by a monoclonal antibody.^[11]^ Serum CA125 levels are typically elevated in most ovarian malignancies and are elevated in many benign diseases too, such as endometriosis and inflammation;^[12]^ therefore, serum CA125 is a specific diagnostic marker for ovarian thecomas. However, the sensitivity of CA125 was not hyperintense, as only 10 patients in our study had elevated CA125, with an incidence rate of 22.2% (10/45). Ovarian thecoma is an ovarian functional tumor; in our study, 20 cases had elevated estrogen levels, and 12 showed clinical manifestations of postmenopausal vaginal bleeding. This study showed that elevated estrogen levels were associated with lesion cystic necrosis (*P* = .013, r = 0.347), indicating a certain relationship between the 2, which could be attributed to lesions with cystic necrosis. Larger lesions produced higher levels of estrogen, a phenomenon that has not been reported by other scholars and needs to be studied further.

In this study, lesions ≥5 cm in size were noted on the left side in 19 cases and on the right side in 17 cases, while those <5 cm in size were noted on the left side in 5 cases and the right side in 4 cases. The differences between the 2 groups were not statistically significant (*P* = .881), indicating that there was no difference in the size and location of the lesion. Thirty five cases showed pelvic effusion (incidence, 77.8%), of which 31 were observed in lesions ≥5 cm in size and 4 in lesions <5 cm in size; the difference between the 2 groups was statistically significant (*P* = .007). When ovarian thecoma is present along with pleural effusion and ascites, it is called Meigs syndrome.^[13]^ Ascites can lead to pleural effusion through the lymphatic vessels and across the chest. The nature of the effusion can be serous or bloody, but without tumor cells. The incidence of Meigs syndrome is generally low. Only 1 patient showed Meigs syndrome (incidence rate, 2.2%[1/45]), indicating that the larger the lesion, the more likely the occurrence of pelvic effusion. It may be that the larger the lesion, the more likely it is to cause obstruction of the lymph nodes or veins. Another likely reason could be that a larger lesion is associated with more estrogen production, which then mimics the symptoms of ovarian hyperstimulation syndrome, thereby increasing the permeability of certain local capillaries. This results in pleural effusion and ascites. There were 6 menopausal patients in the <5 cm group and 30 in the ≥5 cm group (*P* = .264), suggesting that menopause had little effect on tumor size.

In this group of patients, the volume of ovarian thecoma was too large, and the incidence of maximal diameter ≥5 cm was 80.0% (36/45). The reason for the analysis was that the clinical manifestations were not typical. The thecoma was detected in some patients only during physical examination. In other cases, the patient was diagnosed when there several oppressive symptoms, and the lesion when finally detected was too big. Pathologically, an ovarian thecoma is completely enveloped,^[14]^ so MRI images show clear borders. In this study, 43 lesions had a clear boundary, and 2 showed a partially blurred boundary or an unclear boundary between surrounding tissues. Inflammatory cell infiltration around the lesion leads to blurring of the edge of the lesion. In this study, MRI results for ovarian thecoma showed diversity, since T_1_WI showed an isointense signal or slightly hypointense signal. T_2_W1 showed a slightly hyperintense signal, isointense signal, or slightly hypointense signal, mainly a slightly hypointense signal, which is consistent with other scholars reports.^[14,15]^ This finding could be primarily attributed to the fact that tumor cells are fibroblast-like cells that are arranged alternately. The cell bundles are separated by fibrous connective tissue interstitium, and collagen fibers contain less water. However, in DWI, the sequence lesions mostly showed hyperintense signal or mixed hyperintense signal, which was consistent with the results obtained by Li.^[16]^ The reason was that the tumor cells continued to proliferate, and the high cell density allowed the lesion to bind. As tumor cells continue to proliferate, the high cell density either causes the combined water component in the lesions to increase or causes cellular edema inside the tumor tissue. Oh et al found that ovarian thecoma is prone to cystic changes^[17]^ and rarely shows bleeding.^[18]^ This study included 35 patients with cystic lesions, with an incidence rate of 77.8% (35/45). Cystic lesions were seen in 32 of 36 cases with a maximum diameter of ≥5 cm (incidence of cystic change, 88.9% [32/36]) and in 3 of 9 cases with a maximum diameter of <5 cm (incidence, 33.3% [3/9]), and the difference between the 2 groups was statistically significant (*P* < .05). This indicated that the larger the lesion, the higher the incidence of cystic change. The clear margin of 1 cystic lesion was round and eccentric, while 1 case showed bleeding with large lobes and an irregular shape, and 33 cases showed clear boundaries with an irregular shape. The lesions were expressed as a solid, speckled, fissure, or flocculent on T_2_WI hyperintense signal, which showed a flocculation rate of 45.7% (16/35) alone. The change was characterized by flocculosis. In this study, only 1 case of cystic necrosis showed large lobes with hemorrhage, and the remaining lesions showed lobes smaller than half the lesion volume that were mostly located in the central area of the lesion, which was more common in lesions of the ≥5 cm group. The reason for the analysis was that the lesion volume was large, and the arterial blood supply not rich; thus, it was easy for cystic lesions to appear in the presence of ovarian thecoma, but the growth rate of the lesion was slow and the internal structure dense, so the cystic range is small and started from the central area with less blood supply. After the enhanced scan, 44 cases showed mild uneven enhancement in the early stage of enhancement, and the degree of enhancement increased after delay, but it was still lower than normal myometrial enhancement. This pattern of enhancement suggests less blood supply to the tumor, which is comparable to the results obtained by other authors.^[2,19,20]^ The area showing cystic necrosis was not enhanced. One patient presented with obvious enhancement in the arterial, venous and delayed phases of dynamic enhancement scanning. This patient was 32 years old and had a maximum lesion diameter of 3.0 cm. Large sample analysis was needed to determine whether the significant lesion enhancement was correlated with the size and age of the lesion.

MRI imaging diagnoses were scored by 2 hospital diagnostic doctors on the basis of the typical characteristics of the lesions. The lesions were assigned scores from 1 to 6, with higher scores indicating a greater likelihood of ovarian thecoma. For lesions ≥5 cm in this study, the proportion of lesions scoring 6 points was 86.1% (31/36), which was significantly higher than the corresponding proportion (33.3%; 3/9) in the <5 cm group, indicating that larger lesions showed more typical MRI findings, allowing easier diagnosis.

MRI manifestations of ovarian thecoma have certain characteristics, but they should be differentiated from broad ligament fibroids, fallopian tube cancer, and ovarian cancer.

1. Wide ligament fibroids: Occurs in women under 50 years of age, wherein the DWI signal is variable, and the degree of enhancement is similar to normal myometrium, with normal estrogen levels.
2. Fallopian tube cancer: Most of them are “sausage-like” changes, which may be accompanied by different degrees of hydrosalpinx.^[21]^ The form of accumulated water edges is clear.
3. Ovarian cancer: manifested more as an irregular cystic solid mass, blurred borders, enhanced solid components, enhanced abdominal, peritoneal, mesenteric metastasis, with a large ascitic volume.

The clinical significance of correct diagnosis: there are many pathological types of ovarian tumors. Preoperative differential diagnosis has a great influence on the prognosis.^[22]^ Preoperative diagnosis of ovarian thecoma is clinically significant:

Preoperative correct diagnosis can determine the appropriate treatment plan. A small number of ovarian thecomas can be malignant. If misdiagnosed, it may lead to insufficient preparation and imperfect diagnosis and treatment plan, which will affect the prognosis and outcome.

Preoperative diagnosis of ovarian thecoma may prompt the pathologist to use a more complete specimen for testing and subsequent examination to avoid missed diagnosis.

ovarian thecoma belongs to the family of ovarian functional tumors, and some patients may show elevated estrogen levels, which can better explain clinical manifestations such as vaginal bleeding.

Diagnostic experience:

1. Postmenopausal women, unilateral attachment, larger mass, elevated estrogen levels, and clinical vaginal bleeding, often accompanied by the presence of pelvic fluid.
2. In MRI, the edge of the lesion is clear; the cyst is more common; and the cystic range is small, often less than half of the volume of the lesion, and shows a spotted, fissure, or flocculent morphology. Flocculent cysts were the most common lesions measuring ≥5 cm, while speckled cysts were the most common lesions <5 cm in size.

The component showed a hyperintense signal on DWI, and when the lesion is large, it can be a mixed hyperintense signal, with a mildly uneven enhancement that continues to strengthen.

The novel findings of this study were as follows:

1. this study analyzed the size of the lesions and found that the incidence of pelvic fluid and cystic necrosis differed by size, and then further analyzed the morphology and extent of lesion cyst necrosis.
2. The findings showed that elevated estrogen levels are associated with lesion cystic necrosis, which has not been reported in previous literature.
3. In comparison with previous reports, this study had a larger sample and performed a more comprehensive and detailed analysis of ovarian thecomas.

The limitations of this study were as follows:

1. This was a retrospective study. The pathological diagnosis of ovarian thecomas in our study population showed that none of the lesions were malignant.
2. Few lesions were significantly enhanced, and in-depth analysis of these lesions was not performed.

In summary, MRI manifestations of ovarian thecomas have certain characteristics, such as single lesion; clear edge; slight enhancement in dynamic enhancement scans; hyperintense or mixed hyperintense signals for larger lesions on DWI with more combined pelvic effusion and cyst necrosis, small range of cystic necrosis, and mostly flocculent cysts; hyperintense signals for smaller lesions on DWI with mostly speckled morphology in cystic necrosis. Thus, MRI characteristics in combination with patient age and laboratory examination data and other factors can help improve the accuracy of preoperative diagnosis of these tumors.

## Author contributions

**Conceptualization:** Zhi Li, Qingwei Hu, Zhiqin Luo, Wei Zhou, Linghong Xie

**Data curation:** Zhi Li, Qingwei Hu, Zhiqin Luo, Wei Zhou.

**Formal analysis:** Qingwei Hu, Zaixing Deng.

**Investigation:** Zhi Li, Qingwei Hu,Zaixing Deng, Wei Zhou, Linghong Xie.

**Methodology:** Zhi Li,Wei Zhou, Linghong Xie.

**Project administration:** Linghong Xie.

**Resources:** Qingwei Hu,Zaixing Deng, Wei Zhou.

**Supervision:** Linghong Xie.

**Validation:** Zhi Li,Zaixing Deng.

**Visualization:** Zaixing Deng.

**Writing – original draft:** Zhi Li,Zhiqin Luo,Linghong Xie.

**Writing – review & editing:** Zhi Li,Zhiqin Luo,Linghong Xie.
